# Drug Repurposing to Inhibit Histamine *N*-Methyl Transferase

**DOI:** 10.3390/molecules28020576

**Published:** 2023-01-06

**Authors:** Elvia Mera Jiménez, Teresa Żołek, Paola Gabriela Hernández Perez, Rene Miranda Ruvalcaba, María Inés Nicolás-Vázquez, Maricarmen Hernández-Rodríguez

**Affiliations:** 1 Laboratorio de Cultivo Celular, Sección de Posgrado e Investigación, Escuela Superior de Medicina, Instituto Politécnico Nacional, Salvador Díaz Mirón Esq. Plan de San Luis s/n, Casco de Santo Tomas, Miguel Hidalgo, Mexico City 11340, Mexico; 2 Department of Organic and Physical Chemistry, Faculty of Pharmacy, Medical University of Warsaw, Banacha 1, 02-091 Warsaw, Poland; 3 Química Inorgánica-Orgánica del Departamento de Ciencias Químicas, Facultad de Estudios Superiores Cuautitlán Campo 1, UNAM. Av. Primero de Mayo S/N, Sta María Guadalupe las Torres, Cuautitlán Izcalli 54740, Mexico

**Keywords:** drug repurposing, histamine *N*-methyl transferase, computational studies, molecular docking studies, molecular dynamics simulations

## Abstract

Lower activity of the histaminergic system is associated with neurological disorders, including Alzheimer’s disease (AD). Thus, the enhancement of histaminergic neurotransmission by inhibition of histamine *N*-methyl transferase (HNMT), which degrades histamine, appears as an important approach. For this purpose, rigid and flexible molecular docking studies of 185 FDA-approved drugs with the HNMT enzyme were carried out to select two compounds to perform molecular dynamics (MD) simulations to evaluate the binding free energies and stability of the enzyme–drug complexes. Finally, an HNMT inhibition assay was performed to corroborate their effect towards HNMT. Molecular docking studies with HNMT allowed the selection of dihydroergotamine and vilazodone since these molecules showed the lowest Gibbs free energy values. Analysis of the binding mode of vilazodone showed interactions with the binding pocket of HNMT with Glu28, Gln143, and Asn283. In contrast, dihydroergotamine binds to the HNMT active site in a different location, apparently because it is overall the more rigid ligand compared to flexible vilazodone. HNMT inhibitory activity for dihydroergotamine and vilazodone was corroborated (IC_50_ = 72.89 μM and 45.01 μM, respectively) by in vitro assays. Drug repurposing of HNMT was achieved by employing computational studies.

## 1. Introduction

Histamine is a biogenic amine that has a wide range of functions. In the brain, histamine regulates the sleep–wake cycle, sense of reward, emotion, learning, and neuroinflammation [1]. Brain histaminergic neurons origin in the tuberomamillary nucleus and produce histamine by histidine decarboxylase (HDC) employing histidine as a substrate [2]. Histamine effects are a result of their binding to their receptors 1–4 (HR1–HR4), which are G protein-coupled receptors. H1R and H2R are located mainly at the postsynaptic level and mediate excitatory effects [3]. H3R is located on the soma, dendrites, and axons of both histaminergic and non-histaminergic neurons. H3R inhibits the synthesis and release of histamine and other neurotransmitters [4]. In contrast, H4R is expressed in hematopoietic cells and has been associated with inflammatory and immune responses [5]. In the brain, histamine degradation by histamine *N*-methyl transferase (HNMT) is the exclusively known pathway for the ending of histamine effects [6]. Alterations of the histaminergic system have been described in several neurological diseases [7]. Interestingly, decreased histamine levels have been shown in Alzheimer’s disease (AD), depression, and narcolepsy; as a result, the increase in histamine levels represents a new approach [8].

Interfering with neurotransmitter clearance systems is a widely employed approach to treat neurological diseases and allowed the development of acetylcholinesterase inhibitors, tricyclic antidepressants, and selective serotonin re-uptake inhibitors, among others [9]. This evidence highlights that histamine-degrading mechanisms could be a therapeutic approach to develop novel drugs to improve brain functions [10]. Although several molecules have been identified as HNMT inhibitors, its effects in increasing the histamine levels are poor mainly by their low blood–brain penetration, such as amodiaquine [11]. The repositioning of drugs has a great impact on the development of new therapeutics since it allows to identify new uses for an already approved drug, which significantly reduces costs and research time, letting in this sense new treatments to be found for relevant neurological diseases [12].

Related to the above commentaries, the goal of the present research was to perform, in a first instance, rigid and flexible molecular docking studies of 185 FDA-approved drugs with the HNMT enzyme to select two compounds to perform molecular dynamics (MD) simulations to determinate the stability of the enzyme-ligand complexes. Finally HNMT inhibition assay of selected compounds was performed.

## 2. Results

### 2.1. Virtual Screening of Compound against HNMT

A database including 185 FDA-approved drugs employed for the treatment of neurological diseases was constructed, including metoprine as a reference compound. The database includes drugs from several pharmacological classes, including analgesics, anticonvulsants, antiemetics, antiparkinsonians, anxiolytics, sedatives, hypnotics, cholinergic agonists, cholinesterase inhibitors, CNS stimulants, drugs employed in alcohol dependence and antidepressants.

Table S1 in Supplementary Materials shows the drugs name, PubChem CID, and therapeutical use according to the currently described pharmacological effects. Drugs are listed accordingly, with their ∆G values expressed in kcal/mol (from highest to lowest affinity) calculated by rigid docking studies employing AutoDock 4.2. The 3D structure of HNMT was obtained from Protein Data Bank (PDB ID: 2AOV), which has been co-crystalized with metoprine. According to ∆G values, dihydroergotamine (−13.41 kcal/mol), vilazodone (−12.86 kcal/mol), and ergotamine (−12.58 kcal/mol) were selected because they represent the drugs with the highest affinity to HNMT, as shown in Table 1.

**Figure 1:**
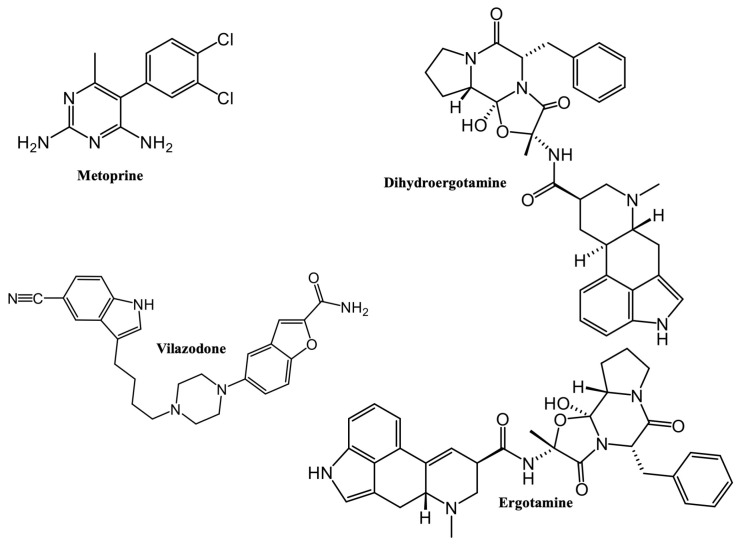
FDA-approved drugs to treat neurological diseases with the highest affinity to HNMT according to docking studies.

From rigid docking studies, it is possible to observe that selected drugs and reference HNMT inhibitor (metoprine) showed interactions with amino acids located in the histamine-binding domain, and particularly with Tyr-146, Tyr-147, Val-173, Trp-179, Trp-183, Cys-196, and Tyr-198, which have been known to play important roles in HNMT inhibition [8]. The structures of selected compounds are showed in Figure 1. As can be seen, dihydroergotamine, ergotamine and vilazodone are bigger compounds with polar substituents in comparison with metoprine. Dihydroergotamine and ergotamine belong to vasoconstrictors alkaloids that are employed to treat migraine [13], while vilazodone is a selective serotonin reuptake inhibitor (SSRI) and partial serotonin receptor agonist employed to treat depressive disorders [14]. Drug–protein complexes of the selected compounds were submitted to flexible docking studies to allow flexibility of amino acid residues and increase the accuracy of computational studies.

### 2.2. Binding Site in Enzyme HNMT by Flexible Docking Studies

HNMT is an enzyme composed of 292 amino acid residues formed by a two-domain structure which includes the methyl donor *S*-(5′-adenosyl)-L-methionine (AdoMet) binding site and the histamine-binding site [15]. From the crystal structure of 2AOV, the histamine-binding domain of HNMT comprises Tyr-15, Phe-19, Phe-22, Glu-28, Gln-143, Tyr-146, Tyr-147, Val-173, Trp-179, Trp-183, Cys-196, Gln-197, Tyr-198, Phe-243, and Glu-246. Analysis of the binding mode of the crystalized metoprine–HNMT complex showed that crucial interactions are represented by a strong hydrogen bonds with two amide nitrogen atoms and one chlorine atom of dichlorophenyl ring of metoprine and amino acid residues Glu-28, Tyr-147 and Gln-197, respectively; in addition, the hydrophobic interaction with two chlorine atoms of dichlorophenyl ring and a methyl group of the pyrimidine ring of metoprine with the amino acid residues Trp-183, Tyr-198, Tyr-146, Trp-179 and Val-173, respectively (Figure S1A in Supplementary Materials). Accordingly, the described pocket was used in the molecular docking procedure, employing the CDOCKER module.

**Figure 2:**
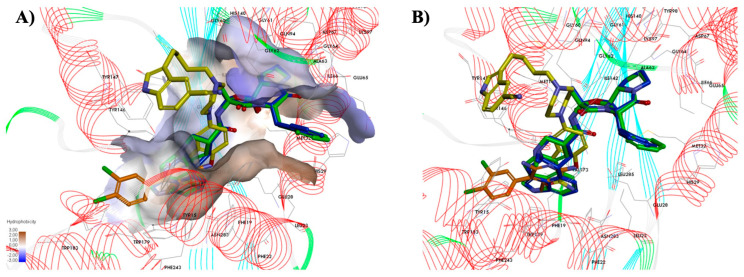
Binding mode of selected compounds with HNMT obtained by flexible docking studies employing CDOCKER. (**A**) The hydrophobic and hydrophilic amino acid residues surrounding the ligands. Surface hydrophobicity was depicted by the shaded colors: brown—the hydrophobic and blue—the lipophilic regions. (**B**) Superposition of compounds: metoprine (orange), dihydroergotamine (blue), vilazodone (yellow), and ergotamine (green) in the histamine-binding site of HNMT.

Initially, the re-docking of metoprine to the binding site of HNMT enzyme was carried out to validate that the reference ligand was docked to the HNMT receptor in the way in which it was experimentally observed. The resulting complex showed ten significant amino acids involved in the interactions: Tyr-15, Glu-28, Tyr-146, Tyr-147, Val-173, Trp-179, Trp-183, Cys-196, and Tyr-198, which are important for HNMT recognition [8] (Figure S1C in Supplementary Materials). The RMSD between the docked and the crystal structure of metoprine was only 0.98 Å (less than 1 Å), which is satisfactory. After that, the selected compounds were docked to the HNMT pocket. The predicted location of the tested compounds with metoprine (as an inhibitor) exhibited a difference in binding mode, which results from significant structural formula differences between the structures of the tested compounds and the reference molecule. Nevertheless, as shown in Figure 2, the benzofuran ring of vilazodone and the indole ring of the ergotamine and dihydroergotamine were similarly located within the hydrophobic active site pocket, in the same place as the pyrimidine ring of metoprine. The remaining molecule elements (aromatic ring, ergoline ring, peptide side chain, and alkyl chain) were the deciding factors showcasing the differences in binding modes with the HNMT enzyme.

### 2.3. Evaluation of Interactions with the Histamine-Binding Site of HNMT by MD Simulations

The MD simulation and binding free energy calculations were performed for a better understanding of the various interactions between the ligand and active site of HNMT. For this reason, vilazodone and dihydroergotamine were selected, in addition to metoprine, to perform MD simulations. The structures of the HNMT–drug complexes obtained by flexible docking studies on HNMT was employed as starting structure for MD simulations. The structures of the complexes were optimized with water molecules and ions. The RMSD value between the average structures of replicate MD simulations, as well as between the ligand starting and average structures, remained at a low level in all cases. The calculated ∆G_bind_ of the three complexes is shown in Table 2.

**Figure 3:**
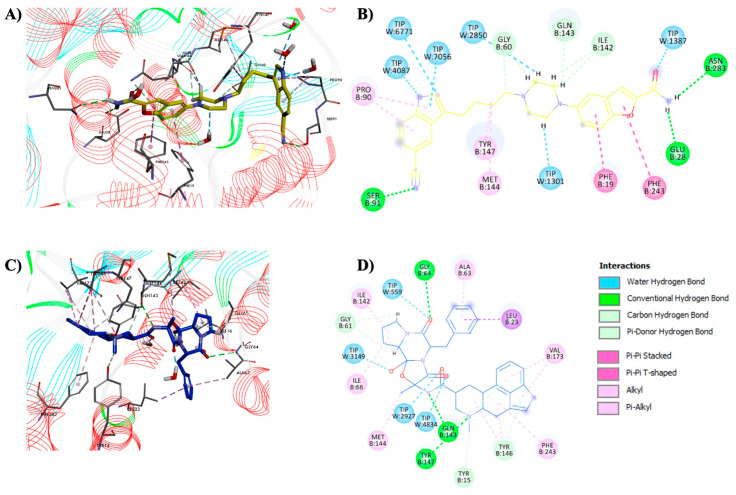
Interactions of the selected compounds with HNMT were obtained by 50 ns of MD simulations. Vilazodone–HNMT complex; binding mode (**A**) and non-bonding interactions (**B**). Dihydroergotamine–HNMT complex; binding mode (**C**) and non-bonding interactions (**D**).

The calculated ∆G_bind_ values of the analyzed complexes showed that ∆G_bind_ (vilazodone) < ∆G_bind_ (dihydroergotamine). The obtained results showed that compounds are effective for targeting HNMT, especially vilazodone, with binding energy like metoprine, the standard HNMT inhibitor. The MD resulting orientations of the tested compounds in the histamine-binding site of HNMT are presented in Figure 3. The poses of the tested compounds showed that they interacted with the binding pocket of a protein target in a similar way to metoprine but the mode to which they bind to HNMT is different.

The interaction of vilazodone showed that the inhibitor bound is suitably for the binding pocket of HNMT (Figure 3A). This compound forms hydrogen bonds with Glu-28, Gly-60, Ser-91, Ile-142, Gln-143 and Asn-283 amino acids through amide and cyano nitrogen atoms and the piperazine ring of vilazodone. It should be noted that three of these residues, Glu-28, Gln-143 and Asn-283, allow the form of direct or water-mediated hydrogen bonds with the best lead molecule metoprine in the crystal structure of HNMT protein (Figure 3B). In addition, important residues such as Phe-19, Phe-243, Tyr-147, Met-144 and Pro-90 interact at each end with the aromatic rings of vilazodone forming hydrophobic interactions. Vilazodone is also highly solvated by water molecules present inside the pocket of HNMT, playing an important role in the binding of vilazodone to HNMT. In contrast to vilazodone, compound dihydroergotamine (IC_50_ = 72.89 µM) binds to the HNMT active site in a different location, apparently because it is overall, the most rigid ligand, in comparison to the flexibility of vilazodone. This could explain the slightly lower affinity of dihydroergotamine for HNMT than vilazodone. It also can be observed that the ergoline ring position of dihydroergotamine causes similar interactions with the enzyme-binding domain as the pyrimidine ring of metoprine (Figure 3C). Dihydroergotamine is surrounded by Tyr-15, Gly-61, Gly-64, Tyr-141, Tyr-146 and Gln-147 of HNMT forming hydrogen bonds with the ligand, and Ile-142, Ala-63, Leu-23, Val-173, Phe-243, Met-144 and Ile-66 creating strong hydrophobic interactions (Figure 3D).

### 2.4. Stability of MD Simulations

**Figure 4:**
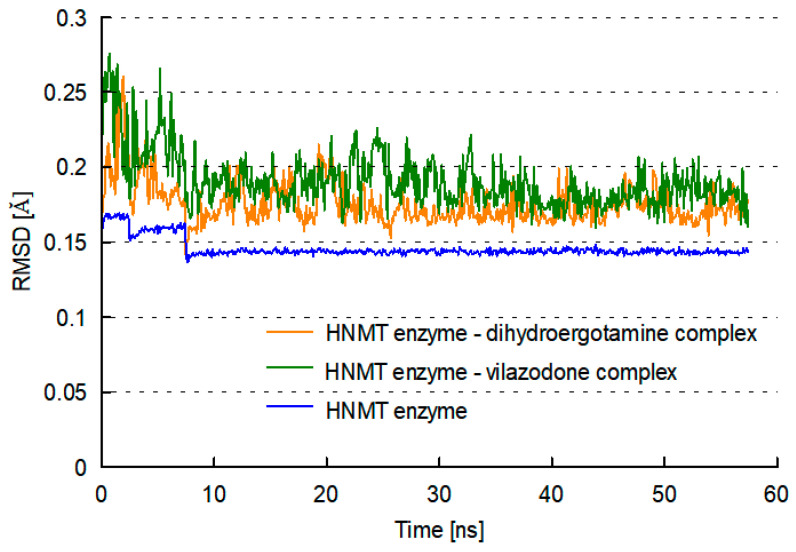
Root-mean-square deviation (RMSD) values for the HNMT–ligand complex obtained during MD simulations; HNMT protein backbone (blue line), HNMT–vilazodone complex (green line) and HNMT–dihydroergotamine complex (orange line).

To investigate the stability of enzyme–ligand interactions during molecular dynamics, we evaluated the structure of the HNMT–vilazodone complex and HNMT–dihydroergotamine complex using the root-mean-square deviation (RMSD) and the root-mean-square fluctuation (RMSF) analysis. The trajectory of the RMSD and the RMSF of the enzyme–ligand complex was monitored using Analyze Trajectory tool of DiscoveryStudio v.21.1 software. The RMSD is the measure of the distance between the enzyme backbones of the superimposed enzyme, to determine the conformational stability of the enzyme backbone and the enzyme–ligand complex. The RMSD enzyme is essentially studied to understand the movements of different atoms in the enzyme when the ligand is present in the active site of the enzyme. The average RMSD values at 300 K over the course of the 50 ns were outlined for the trajectory structures of all tested complexes (Figure 4). As the plots show, the RMSDs of each system tend to converge after 35 ns simulation time, indicating the system is stable and equilibrated. All systems show very small RMSDs suggesting that they are similar in conformation to the crystal structure. The RMSD plot for the vilazodone–HNMT complex was observed to be in the range of 0.15–0.28 Å while for the complex with dihydroergotamine, the value was in the range of 0.14–0.26 Å. Therefore, these results clearly indicate that the presence of vilazodone and dihydroergotamine in the active site of the enzyme has maintained the stability of the enzyme throughout the simulation period, but the stability was better in the presence of dihydroergotamine. This fact must be due since dihydroergotamine is a more rigid ligand compared to flexible vilazodone. These results additionally imply that the explicit solvation system is the correct choice for the investigations of ligand–HNMT binding processes.

**Figure 5:**
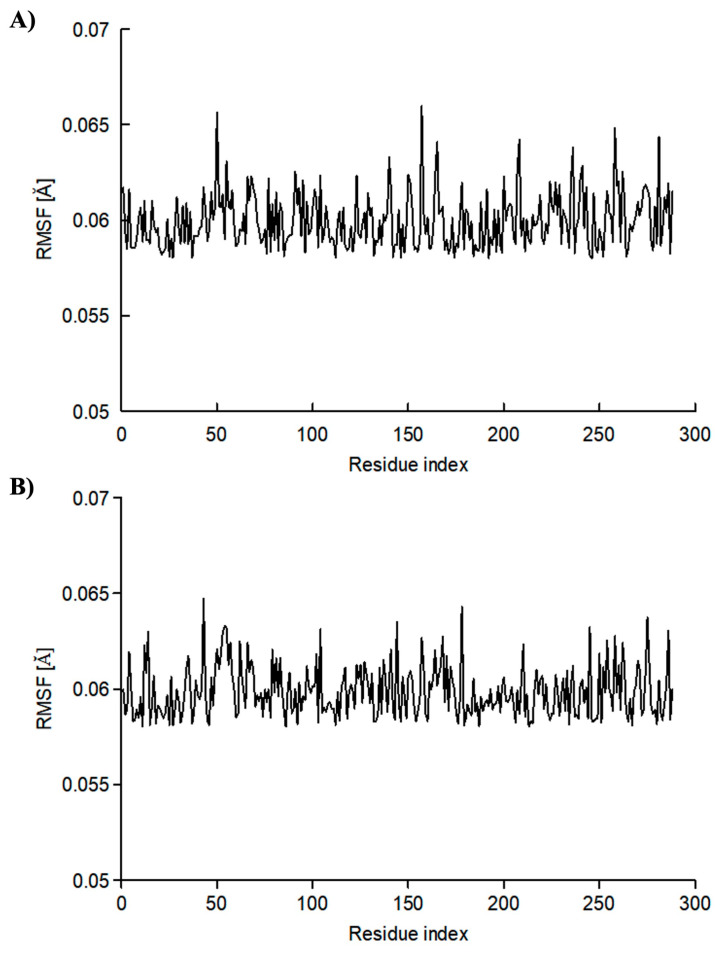
Residue-wise root-mean-square fluctuation (RMSF) value for the HNMT–ligand complex obtained during MD simulations; HNMT–vilazodone complex (**A**) and HNMT–dihydroergotamine complex (**B**).

In addition, the RMSF was calculated to predict the structural integrity of the HNMT backbone and ligand (vilazodone and dihydroergotamine) complex. Figure 5 shows the analysis values of the residue-wise RMSF when the HNMT enzyme is bound with vilazodone and dihydroergotamine, respectively. In this plot, each peak indicates the protein area that fluctuates the most during the MD simulation.

Both figures show almost similar pattern peaks in which the higher peaks correspond to loop regions identified from the MD simulation trajectories. The lower value of the RMSF indicates the stability of ligands binding to the HNMT enzyme. For all the amino acid residues in the enzyme, the RMSF was below 0.07 Å when it was complexed with vilazodone (Figure 5A) and the RMSF was below 0.065 Å when it was complexed with dihydroergotamine (Figure 5B).

### 2.5. HNMT Inhibition Assay

**Figure 6:**
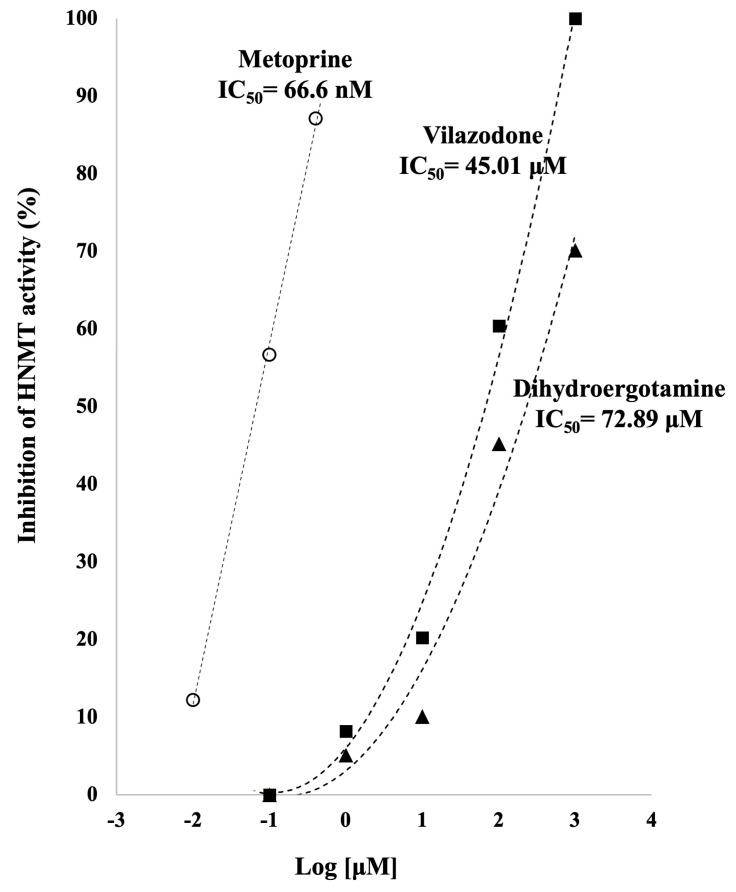
Quantitative HNMT inhibition by metoprine (66.6 nM), dihydroergotamine (72.89 µM) and vilazodone (45.01 µM). These results demonstrate that vilazodone and dihydroergotamine are inhibitors of HNMT. The activity of HNMT in the absence of compounds was considered 100% of activity. Values on the graph represent the mean values. The concentration–response curve was obtained and the IC_50_ values were estimated for all compounds through linear regression analysis.

To corroborate the results obtained by computational studies, the HNMT activity in presence of vilazodone, dihydroergotamine, and metoprine was evaluated according to manufacturer instructions. As can be seen in Figure 6, all the studied compounds inhibit HNMT; however, selected drugs showed a lower potency in comparison with metoprine to inhibit HNMT. The IC_50_ calculated by metoprine (66.66 nM) was similar to previous reported IC_50_ values [16]. HNMT inhibition exhibited by vilazodone (IC_50_ = 45.01 μM) and dihydroergotamine (IC_50_ = 72.89 µM) was in the micromolar range.

## 3. Discussion

The complexity of the pathophysiology and the lack of concrete evidence of molecular targets are the major hurdles to the development of a new drug to treat AD. The high discontinuation rate of many advanced drugs in the clinical phases makes the process of new discovery very expensive [17]. In addition, although there has been much effort to further the knowledge of the pathological mechanism of AD and advances in technology, the development of novel drugs is still a large process with a great probability of failing [18]. Therefore, repurposing of ‘old’ drugs is becoming increasingly attractive, as it involves the use of agents with fewer side effects and potentially shorter development times [19].

Recently, enhancing histamine neurotransmission by inhibition of their catabolic enzyme HNMT has been proposed as a potential therapeutical strategy for AD patients [8], due to their effects on cognitive functions [20], neuroplasticity [21], neurogenesis [22], and the degradation of the amyloid beta (Aβ) peptide [23]. Consequently, a combination of computational approaches during drug repurposing offers benefits in AD drug development. Docking of small compounds to receptor-binding sites and estimation of the binding affinity of the complex allows the selection of promising compounds [24]. Employing rigid docking studies, a virtual screening was performed to select FDA-approved drugs with the highest affinity to HNMT. In this way, ergotamine, dihydroergotamine and vilazodone were selected from a database of 185 FDA drugs employed to treat neurological diseases. Only drugs employed to treat neurological diseases were considered to guarantee that drugs cross the blood–brain barrier (BBB). Afterward, flexible docking studies were conducted to determine the protein–ligand interactions and to improve the performance of computational calculations [25]. Ergotamine and dihydroergotamine are commonly used in the treatment of migraine [26], while vilazodone is employed to treat depression [14]. Since there are only slight differences between ergotamine and dihydroergotamine interactions with HNMT in addition to the fewer side-effects for dihydroergotamine than ergotamine [27], only dihydroergotamine was submitted to MD simulations.

Analysis of the binding mode of vilazodone obtained by MD simulations showed that the inhibitor bounds well to the binding pocket of HNMT by forming hydrogen bonds with Glu-28, Gly-60, Ser-91, Ile-142, Gln-143 and Asn-283 amino acids through their amide and cyano nitrogen atoms with the piperazine ring. This fact is important because the formation of hydrogen bonds between a polar atom of the inhibitors with Glu28, Gln143, and Asn283 of HNMT has been reported [28]. In addition, vilazodone establishes a set of hydrophobic interactions with Phe-19, Phe-243, Tyr-147, Met-144, and Pro-90. Vilazodone is also highly solubilized by water molecules located in the pocket of HNMT, which may play an important role in the binding of vilazodone to HNMT. In contrast, dihydroergotamine binds to the active site of HNMT at a different location, apparently because it is the more rigid ligand overall compared to the flexible vilazodone. In contrast, dihydroergotamine did not establish hydrogen bonds with Glu28, Gln143, and Asn283 of HNMT. In addition, all systems show very small RMSDs that clearly indicate that the presence of vilazodone and dihydroergotamine in the active site of the enzyme has maintained the stability of the enzyme throughout the simulation period.

In vitro assays corroborated that metoprine showed HNMT inhibition in the nanomolar range, as previously reported [28]. In contrast, vilazodone and dihydroergotamine inhibit HNMT in the micromolar range (IC_50_ = 45.01 μM and 72.89 μM, respectively), thus representing promising compounds.

Metoprine is an HNMT inhibitor widely employed to demonstrate the effects of the increase in histamine levels in the brain. Interestingly, it has been shown that HNMT inhibition by metoprine ameliorated memory impairments in male Sprague Dawley mice [29]. However, their therapeutic application is limited due to their adverse effects. By clinical trials, it has been shown that metoprine produces cutaneous, gastrointestinal, and hematological toxicities which have been related to their antifolate activity [30,31]. In addition, metoprine binds to more than 87% of serum proteins and has a plasma half-life of 216 h [32]. In comparison, HNMT inhibitors repurposed in the present work show advantages in contrast with metoprine. The half-life of vilazodone after oral administration is approximately 25 h and shows a bioavailability of 72%. Their adverse effects are mainly diarrhea, headache, nausea, and dizziness [33]. In contrast, the absolute bioavailability of dihydroergotamine administered by the intramuscular route is 100%. However, an intranasal route can be employed with a bioavailability of approximately 40%. The half-life of dihydroergotamine depends on the route of administration but is approximately 13 h. The adverse effects of dihydroergotamine are infrequent—some of them are nausea, vomiting, muscle pain, and numbness of the fingers and toes [34].

Although oral administration of dihydroergotamine to adult male Wistar Albino Glaxo did not influence the learning processes, dihydroergotamine treatment produced changes in the concentration of monoaminergic neurotransmitters and their metabolites in the prefrontal cortex, striatum, cerebellum, medulla oblongata and spinal cord [35]. In a similar way, although vilazodone has not shown improvements in visuospatial memory in healthy middle-aged female mice, its effects on animal models of cognitive impairment have not been explored [36]. Furthermore, it is important to highlight that vilazodone does not impair cognition, despite most current antidepressants producing memory impairment as a side effect [37].

## 4. Materials and Methods

### 4.1. Selection of Dataset

A set of 185 compounds with diverse structures were collected from a database of FDA-approved drugs employed to treat neurological diseases, including metoprine as a controlled drug. Drugs were obtained from the Drugs Database (https://www.drugs.com/, accessed on 24 January 2022). The three-dimensional structure in .sdf format of selected compounds was obtained from PubChem (https://pubchem.ncbi.nlm.nih.gov, accessed on 18 February 2022) and converted to .pdb format employing Avogadro software (https://avogadro.cc/, accessed on 5 March 2022). The three-dimensional structure of the HNMT enzyme was accessed from RCSB Protein Data Bank (www.rcsb.org, accessed on 24 April 2022) with PDB ID: 2AOV. All ligands, inorganic ions, and solvent molecules that were present in the HNMT original structure were manually removed, and hydrogen atoms were added using the graphical interface of AutoDock 4.2 [38]. The crystal structure of HNMT was used for molecular docking and a search of the active site cavity was performed based on the metoprine inhibitor.

### 4.2. HNMT–Ligand Docking Study

The molecular docking experiments were employed as the elementary method to find the appropriate site for the interaction of the studied molecules with the HNMT target. In a first step, a molecular docking method was used to perform so-called structure-based virtual screening to identify active compounds by filtering out those that do not fit into the binding site of the enzyme. Docking studies were conducted with AutoDock 4.2 software. To check the selected parameters of the AutoDock 4.2 program for HNMT docking, the metoprine inhibitor structure was sketched and re-docked with the HNMT crystal structure and the root-mean-square deviation (RMSD) was calculated between the docked pose and the bound conformation of the crystal metoprine. The final hits retrieved from the database using virtual screening were docked to the active site of the HNMT enzyme. A grid-based procedure was utilized to prepare the structural inputs and define all the binding sites. A rectangular lattice (70 × 70 × 70 Å) with points separated by 0.375 Å was centered on the active site of HNMT (near Glu28, Gln143, and Asn283 residues). All docking simulations were conducted using the hybrid Lamarckian genetic algorithm with an initial population of 100 randomly placed individuals and a maximum of 1.0 × 10^7^ energy evaluations. All other parameters were maintained at their default settings. The resulting docked orientation within an RMSD of 0.5 Å was clustered together. The lowest energy cluster for each ligand was subjected to further free energy and binding geometry analysis, as previously reported [39].

In a second stage, four selected compounds (dihydroergotamine, vilazodone, and ergotamine), including metoprine (reference inhibitor) (Figure 1), were used employing an automated flexible docking protocol as part of the DiscoveryStudio v.21.1 BIOVIA visual interface [40], because AutoDock 4.2 software assumes a rigid enzyme [41], which may affect its accuracy in posing and scoring docked ligands in further research. The geometries of chosen compounds were optimized using the density functional theory (DFT) with the B3LYP/6-311G(d,p) hybrid functional, as implemented in Gaussian 16 [42]. ESP-atomic partial charges on all atoms were computed using the Breneman model reproducing the molecular electrostatic potential [43]. Optimized compounds were imported into the CDOCKER module in DiscoveryStudio v.21.1 software, which relies on the CHARMm force field [44]. The binding site was defined with a radius of 18 Å around the ligand present in the X-ray structure of the HNMT enzyme. The best-fitted conformational poses of each compound were generated and analyzed based on the docking scores (CDOCKER interaction energy). The number of starting random conformations and number of rotated ligand orientations to refine for each of the conformations for 1000 dynamics steps were set to thirty. Moreover, for annealing refinement, the number of heating steps was 2000, while the number of cooling sets was set to 5000. The best-docked poses were selected and then the molecular dynamics (MD) simulations were performed to improve the structural reliability of the ligand–HNMT complexes.

### 4.3. Interaction with HNMT: Molecular Dynamics and Binding Free Energy Calculations

The MD simulations analysis was carried out to find the interaction of enzyme–ligand stability. All MD simulations were run in the CHARMm force field implemented in the module of DiscoveryStudio v.21.1 software. Each model of the HNMT–ligand complex was inserted into a cubic box of water molecules (TIP3P models) [45] extending up to 5 Å from any solute atom. Counterions (Na^+^, Cl^−^) were randomly added to each complex at a concentration of ~0.15 M, like physiological conditions using the solvation module of DiscoveryStudio v.21.1 software. All energy minimization and MD simulations were performed using the Particle Mesh Ewald (PME) algorithm [46] for proper treatment of electrostatic interactions [47] and periodic boundary conditions. Prior to MD simulations, all systems were minimized based on the steepest descent method with 2500 steps followed by 5000 conjugate gradient energy-minimization steps (until the RMS gradient of the structure was below 0.01 kcal/mol·Å) with an applied restraint potential started from 10 kcal/mol·Å^2^ to 1 kcal/mol·Å^2^. The conjugate gradient algorithm without restraint was further carried out with an additional full minimization of 1000 steps. A gradual heating MD simulation from 50 to 300 K was executed for 100 ps. Following the heating, an equilibration estimating 100 ps of each system was conducted (the operating temperature was kept constant at 300 K). In the stages of heating and equilibration, the enzyme was fixed with a force constant of 1 kcal mol^−1^ Å^−2^. The energy-minimized system was further analyzed with NVT and then NPT ensemble for 100 ps simulations at 300 K. The final production MD runs were performed for 50 ns (2 fs per step) with periodic boundary conditions keeping the temperature of the system at 300 K. The trajectories from the MD simulations were saved for every 50 ps intervals for analyses of the root-mean-square deviation (RMSD) and the mean square fluctuation (RMSF) as well as the enzyme–ligand contacts.

The stability of the HNMT–ligand complexes is reflected in the binding free energies (∆G_bind_) calculated by the Molecular Mechanics Poisson–Boltzmann Surface Area (MM-PBSA) method [48] in DiscoveryStudio v.21.1 software. The ∆G_bind_ of tested compounds to HNMT protein was calculated using the following equation: ∆G_bind_ = G_HNMT–ligand_ − G_HMNT_ − G_ligand_, where G_HNMT–ligand_ is the free energy of complex, G_HNMT_ is the free energy of HNMT enzyme and G_ligand_ is free energy tested compounds. Binding free energy was calculated based on the average structures obtained from the 50 ns of MD trajectories.

### 4.4. The HNMT Inhibition Assay

The HNMT inhibition assay was performed employing Recombinant Human Histamine *N*-Methyltransferase/HNMT (rhHNMT) (R&D systems, Catalog number: 7637-MT) according to the instructions provided by the manufacturer in triplicate assays. Histamine methylation by HNMT is evidenced by increased fluorescence at excitation and emission wavelengths of 380 and 445 nm, respectively, thus the HNMT inhibition is demonstrated by the decrease in fluorescence. Vilazodone and dihydroergotamine were evaluated as HNMT inhibitors at crescent concentrations (0.001, 0.1, 1, 10, and 100 μM), employing metoprine (50, 100, and 200 nM) as a reference compound. The percentage of HNMT activity was calculated considering the fluorescence of the enzyme without inhibitor as 100% of activity.

## 5. Conclusions

By employing computational studies, virtual screening towards HNMT of 185 FDA-approved drugs for the treatment of neurological diseases was analyzed. From this group of drugs, we were able to identify potential drugs that could inhibit the function of HNMT. Employing a combination of rigid and flexible docking studies, in addition to MD simulations, dihydroergotamine and vilazodone were selected. The inhibition of HNMT by in vitro assays was corroborated by dihydroergotamine and vilazodone in the micromolar range, being the highest for vilazodone. Therefore, the evaluation of vilazodone and dihydroergotamine on models of cognitive impairment needs to be addressed.

## Figures and Tables

**Table 1:** FDA-approved drugs with the lowest Gibbs free energy (∆G) to HNMT and the amino acid residues which drives their interaction, obtained by rigid docking studies. Metoprine was included as a reference compound.

∆G (kcal/mol)	Drug	Amino Acid Residues
−13.41	Dihydroergotamine	Glu-89, Gln-94, Tyr-147, Tyr-146, Cys-196, Gln-197, Phe-19, Phe-9, Val-173, Phe-22, Phe-243, Trp-183, Tyr-198.
−12.86	Vilazodone	Leu-8, Phe-9, Glu-246, Phe-243, Trp-179, Tyr-147, Pro-191, Asp-193, Trp-183, Cys-196, Tyr-198, Cys-196, Gln-197, Val-173, Tyr-146.
−12.58	Ergotamine	Glu-89, Gln-94, Tyr-147, Cys-196, Gln-197, Phe-9, Gln-143, Tyr-146, Phe-19, Phe-22, Phe-243, Trp-183, Tyr-198.
−9.08	Metoprine	Tyr-147, Phe-9, Cys-196, Tyr-146, Tyr-198, Gln-143, Trp-183, Val-173, Trp-179, Glu-28, Asn-283.

**Table 2:** Theoretical free energy of binding to the HNMT enzyme of dihydroergotamine, vilazodone, and metoprine obtained by MD simulations.

Complex	∆G_bind_ [kcal/mol]
Dihydroergotamine	−55.45
Vilazodone	−89.42
Metoprine	−98.61

## Data Availability

The datasets generated during and/or analyzed during the current study are available upon request to dra.hernandez.ipn@gmail.com and nicovain@yahoo.com.mx.

## Supplementary Materials

The following supporting information can be downloaded at: https://www.mdpi.com/article/10.3390/molecules28020576/s1, Figure S1: Binding mode of metoprine in the crystal structure of HNMT protein (PDB ID: 2AOV) before re-docking (red color) and after re-docking (orange color). The RMSD between the re-docking pose and the crystallographic structure was 0.98 Å. Table S1: FDA-approved drugs selected for this study which are used to treat neurological disorders.
