# Supplementary material for: Drug Repurposing to Inhibit Histamine N-Methyl Transferase
Source: Molecules. 2023 Jan 6;28(2):576. doi: 10.3390/molecules28020576 (PMC9867436; doi:10.3390/molecules28020576)
Supplement: Supplementary file 1 [file molecules-28-00576-s001.zip › molecules-2114106-supplementary.pdf]

Supplementary materials

Drug repurposing to inhibit brain Histamine N-Methyl Transferase

Elvia Mera Jiménez, Teresa Żołek, Paola Gabriela Hernández Perez, Rene Miranda Ruvalcaba, María Inés Nicolás-Vázquez, Maricarmen Hernández-Rodríguez

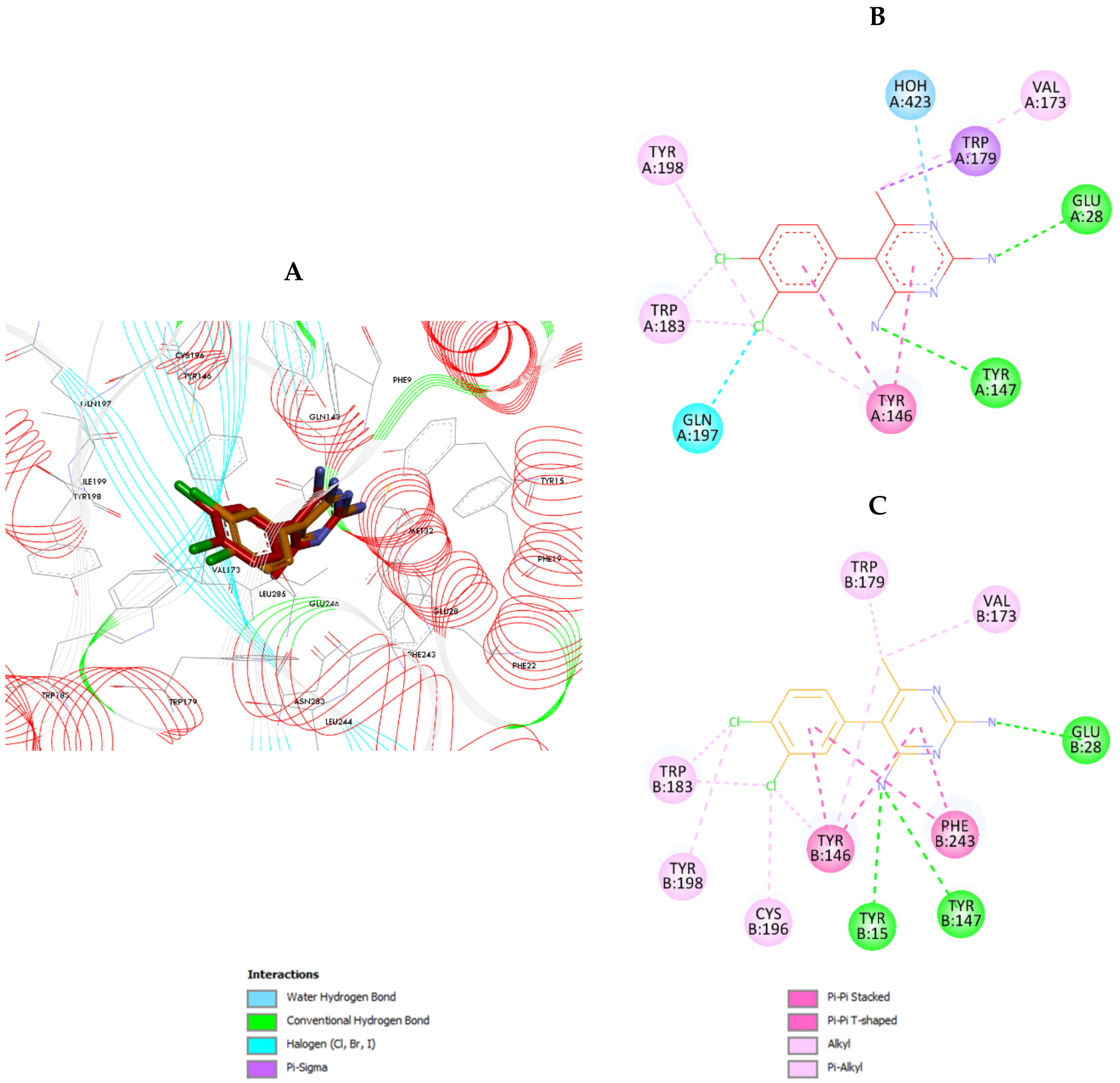

Figure S1. Binding mode of metoprine in the crystal structure of HNMT protein (PDB ID: 2AOV) before re-docking (red colour) and after re-docking (orange colour). (RMSD between the redocking pose and the crystallographic structure was 0.98 Å).

Table S1. FDA-approved drugs employed to treat neurological disorders employed in the present study and their binding free energies (ΔG) towards HNMT obtained by rigid docking studies employing AutoDock 4.2.

ΔG (kcal/mol)	Drug	Molecular weight (g/mol)	PubChem CID	Therapeutic indication
-13.41	Dihydroergotamine	583.7	10531	Antimigraine
-12.86	Vilazodone	441.5	6918314	Antidepressants
-12.58	Ergotamine	581.7	8223	Antimigraine
-12.27	Nurtec ODT (rimegepant sulfate)	1221.2	51049968	CGRP inhibitors
-11.98	Meclizine	390.9	4034	Antiemetic/antivertigo agents
-11.83	Ubrelvy (ubrogepant)	549.5	68748835	CGRP inhibitors
-11.60	Qulipta (atogepant)	603.5	72163100	CGRP inhibitors
-11.59	Valbenazine	418.6	24795069	VMAT2 inhibitors
-11.52	Rolapitant	500.5	10311306	Antiemetic/antivertigo agents
-11.48	Buspirone	385.5	2477	Miscellaneous anxiolytics, sedatives and hypnotics
-11.45	Perampanel	349.4	9924495	Anticonvulsivants
-11.41	Eletriptan	382.5	77993	Antimigraine
-11.41	Nefazodone	470.0	4449	Antidepressants
-11.41	Amodiaquine	355.9	2165	Antidepressants
-11.19	Benztropine	307.4	1201549	Antiparkinson agents
-11.14	Oliceridine	386.6	66553195	Narcotic analgesics
-11.13	Butorphanol	327.5	5361092	Narcotic analgesics
-11.11	Thiethylperazine	399.6	5440	Antiemetic/antivertigo agents
-11.08	Suvorexant	450.9	24965990	Miscellaneous anxiolytics, sedatives and hypnotics
-10.98	Perphenazine	404	4748	Antiemetic/antivertigo agents
-10.95	Prochlorperazine	373.9	4917	Antiemetic/antivertigo agents
-10.86	Dolasetron	324.4	3033818	Antiemetic/antivertigo agents
-10.69	Nabilone	372.5	5284592	Antiemetic/antivertigo agents
-10.68	Biperiden	311.5	2381	Antiparkinson agents
-10.67	Palonosetron	296.4	6337614	Antiemetic/antivertigo agents
-10.64	Donepezil	379.5	3152	Cholinesterase inhibitors
-10.55	Alfentanil	416.5	51263	Narcotic analgesics
-10.55	Fluraepam	387.9	3393	Anxiolytics, sedatives, and hypnotics
-10.51	Fentanyl	336.5	3345	Narcotic analgesics
-10.46	Fosaprepitant	614.4	135413538	Antiemetic/antivertigo agents
-10.41	Trihexyphenidyl	301.5	5572	Antiparkinson agents
-10.25	Tiagabine	375.6	60648	Anticonvulsivants
-10.20	Dronabinol	314.5	16078	Antiemetic/antivertigo agents
-10.16	Nalbuphine	357.4	5311304	Narcotic analgesics

-10.13	Maprotiline	277.4	4011	Antidepressants
-10.08	Sufentanil	386.6	41693	Narcotic analgesics
-10.06	Vortioxetine	298.4	9966051	Antidepressants
-10.05	Pentazocine	285.4	441278	Narcotic analgesics
-10.02	Naltrexone	341.4	5360515	Anorexiants
-10.01	Escitalopram	324.4	146570	Antidepressants
-10.00	Lisdexamfetamine	263.38	11597698	CNS stimulants
-9.96	Citalopram	324.4	2771	Antidepressants
-9.96	Sertraline	306.2	68617	Antidepressants
-9.94	Nortriptyline	263.4	4543	Antidepressants
-9.93	Procyclidine	287.4	4919	Antiparkinson agents
-9.93	Trimipramine	294.4	5584	Antidepressants
-9.92	Rotigotine	315.5	59227	Antiparkinson agents
-9.91	Granisetron	312.4	5284566	Antiemetic/antivertigo agents
-9.91	Chlordiazepoxide	299.75	2712	Anxiolytics, sedatives, and hypnotics
-9.88	Triazolam	343.2	5556	Anxiolytics, sedatives, and hypnotics
-9.79	Protriptyline	263.4	4976	Antidepressants
-9.78	Trazodone	371.9	5533	Antidepressants
-9.76	Aprepitant	534.4	135413536	Antiemetic/antivertigo agents
-9.75	Paroxetine	329.4	43815	Antidepressants
-9.74	Amitriptyline	277.4	2160	Antidepressants
-9.72	Lasmiditan	377.4	4440	Antimigraine
-9.71	Doxepin	279.4	667477	Antidepressants
-9.68	Methadone	309.4	4095	Narcotic analgesics
-9.68	Amoxapine	313.8	2170	Antidepressants
-9.67	Almotriptan	335.5	123606	Antimigraine
-9.63	Levorphanol	257.37	5359272	Narcotic analgesics
-9.63	Cannabidiol	314.5	644019	Anticonvulsivants
-9.57	Hydrocodone	299.4	5284569	Narcotic analgesics
-9.52	Frovatriptan	243.3	77992	Antimigraine
-9.49	Oxycodone	315.4	5284603	Narcotic analgesics
-9.47	Eszopiclone	388.8	969472	Miscellaneous anxiolytics, sedatives and hypnotics
-9.46	Alprazolam	308.8	2118	Anxiolytics, sedatives, and hypnotics
-9.46	Tetrabenazine	317.4	6018	VMAT2 inhibitors
-9.40	Trimethobenzamide	388.5	5577	Antiemetic/antivertigo agents
-9.39	Remifentanil	376.4	60815	Narcotic analgesics
-9.39	Scopolamine	303.35	3000322	Antiemetic/antivertigo agents
-9.39	Deutetrabenazine	323.5	73437646	VMAT2 inhibitors
-9.39	Clomipramine	314.9	2801	Antidepressants
-9.38	Propoxyphene	339.5	10100	Narcotic analgesics
-9.34	Naratriptan	335.5		Antimigraine

-9.34	Fosphenytoin	362.27	56339	Anticonvulsivants
-9.31	Galantamine	287.35	9651	Cholinesterase inhibitors
-9.30	Duloxetine	297.4	60835	Antidepressants
-9.29	Diphenhydramine	255.35	3100	Antidepressants
-9.24	Zaleplon	305.33	5719	Miscellaneous anxiolytics, sedatives and hypnotics
-9.17	Zolpidem	307.4	5732	Miscellaneous anxiolytics, sedatives and hypnotics
-9.17	Mirtazapine	265.35	4205	Antidepressants
-9.16	Lorazepam	321.2	3958	Anticonvulsivants
-9.13	Hydromorphone	285.34	5284570	Narcotic analgesics
-9.13	Istradefylline	384.4	5311037	Antiparkinson agents
-9.08	Metoprine	269.13	24466	Antidepressants
-9.07	Imipramine	280.4	3696	Antidepressants
-9.06	Clonazepam	315.71	2802	Anticonvulsivants
-9.06	Desipramine	266.4	2995	Antidepressants
-9.05	Zolmitriptan	287.36	60857	Antimigraine
-9.05	Ondansetron	293.4	4595	Antiemetic/antivertigo agents
-9.03	Morphine	285.34	5288826	Narcotic analgesics
-9.01	Amisulpride	369.5	2159	Antiemetic/antivertigo agents
-8.95	Temazepam	300.74	5391	Anxiolytics, sedatives, and hypnotics
-8.95	Armodafinil	273.4	9690109	CNS stimulants
-8.86	Sumatriptan	295.4	5358	Antimigraine
-8.85	Carbamazepine	236.27	2554	Anticonvulsivants
-8.85	Oxcarbazepine	252.27	34312	Anticonvulsivants
-8.84	Promethazine	284.4	4927	Antiemetic/antivertigo agents
-8.82	Oxymorphone	301.34	5284604	Narcotic analgesics
-8.81	Modafinil	273.4	4236	CNS stimulants
-8.80	Ramelteon	259.34	208902	Miscellaneous anxiolytics, sedatives and hypnotics
-8.77	Chlorpromazine	318.9	2726	Antiemetic/antivertigo agents
-8.74	Topiramate	339.36	5284627	Anorexiants
-8.71	Clobazam	300.74	2789	Anticonvulsivants
-8.64	Phenytoin	252.27	1775	Anticonvulsivants
-8.61	Memantine	179.3	4054	Cholinesterase inhibitors
-8.56	Sibutramine	279.8	5210	Anorexiants
-8.56	Diazepam	284.74	3016	Anticonvulsivants
-8.55	Doxylamine	270.37	3162	Antiemetic/antivertigo agents
-8.53	Doxylamine	270.37	3162	Miscellaneous anxiolytics, sedatives and hypnotics
-8.51	Atomoxetine	255.35	54841	CNS stimulants

-8.50	Atomoxetine	255.35	54841	Adrenergic uptake inhibitors for ADHD
-8.48	Venlafaxine	277.4	5656	Antidepressants
-8.46	Fluvoxamine	318.33	5324346	Antidepressants
-8.42	Eslicarbazepine	254.28	9881504	Anticonvulsivants
-8.40	Diphenhydramine	255.35	3100	Antiemetic/antivertigo agents
-8.40	Fluoxetine	309.33	3386	Antidepressants
-8.37	Desvenlafaxine	263.37	125017	Antidepressants
-8.27	Levomilnacipran	246.35	6917779	Antidepressants
-8.25	Clorazepate	314.72	2809	Anxiolytics, sedatives, and hypnotics
-8.22	Rizatriptan	269.34	5078	Antimigraine
-8.13	Ropinirole	260.37	5095	Antiparkinson agents
-8.10	Benzphetamine	239.35	5311017	Anorexiants
-8.07	Tramadol	263.37	33741	Narcotic analgesics
-8.05	Tasimelteon	245.32	10220503	Miscellaneous anxiolytics, sedatives and hypnotics
-8.01	Dexmethylphenidate	233.31	154101	CNS stimulants
-7.98	Cenobamate	267.67	11962412	Anticonvulsivants
-7.95	Lorcaserin	195.69	11658860	Anorexiants
-7.88	Meperidine	247.33	4058	Narcotic analgesics
-7.88	Retigabine	303.33	121892	Anticonvulsivants
-7.86	Methylphenidate	233.31	4158	CNS stimulants
-7.70	Buprenorphine	467.6	644073	Narcotic analgesics
-7.70	Stiripentol	234.29	5311454	Anticonvulsivants
-7.67	Tacrine	198.26	1935	Cholinesterase inhibitors
-7.61	Tapentadol	221.34	9838022	Narcotic analgesics
-7.60	Lamotrigine	256.09	3878	Antiemetic/antivertigo agents
-7.58	Mephobarbital	246.26	8271	Anticonvulsivants
-7.53	Bupropion	239.74	444	Anorexiants
-7.53	Isocarboxazid	231.25	3759	Antidepressants
-7.47	Phenobarbital	232.23	4763	Anticonvulsivants
-7.44	Viloxazine	237.29	5666	Adrenergic uptake inhibitors for ADHD
-7.37	Gabapentin enacarbil	329.39	9883933	Anticonvulsivants
-7.35	Felbamate	238.24	3331	Anticonvulsivants
-7.35	Rivastigmine	250.34	77991	Cholinesterase inhibitors
-7.30	Pramipexole	211.33	119570	Antiparkinson agents
-7.25	Melatonin	232.28	896	Miscellaneous anxiolytics, sedatives and hypnotics
-7.23	Metoclopramide	299.79	4168	Antiemetic/antivertigo agents
-7.19	Tacrine	198.26	1935	Antidepressants
-7.05	Phentermine	149.23	4771	Anorexiants
-7.05	Secobarbital	238.28	5193	Anxiolytics, sedatives, and hypnotics
-7.01	Amobarbital	226.27	2164	Anxiolytics, sedatives, and hypnotics

-6.86	Methsuximide	203.24	6476	Anticonvulsivants
-6.84	Mephenytoin	218.25	4060	Anticonvulsivants
-6.83	Lacosamide	250.29	219078	Anticonvulsivants
-6.80	Rufinamide	238.19	129228	Anticonvulsivants
-6.78	Primidone	218.25	4909	Anticonvulsivants
-6.76	Phendimetrazine	191.27	30487	Anorexiants
-6.75	Brivaracetam	212.29	9837243	Anticonvulsivants
-6.74	Pilocarpine	208.26	5910	Cholinergyc agonist
-6.73	Tranylcypromine	133.19	19493	Antidepressants
-6.70	Zonisamide	212.23	5734	Anticonvulsivants
-6.65	Cevimeline	199.32	83898	Cholinergyc agonist
-6.65	Dextroamphetamine	135.21	5826	CNS stimulants
-6.62	Selegiline	187.28	26757	Antiparkinson agents
-6.61	Fenfluramine	231.26	3337	Anticonvulsivants
-6.52	Ethotoin	204.22	3292	Anticonvulsivants
-6.52	Tryptophan	204.22	6305	Miscellaneous anxiolytics, sedatives and hypnotics
-6.46	Acetazolamida	222.3	1986	Anticonvulsivants
-6.45	Butabarbital	212.25	2479	Anxiolytics, sedatives, and hypnotics
-6.44	Amphetamine	135.21	3007	CNS stimulants
-6.42	Diethylpropion	205.3	7029	Anorexiants
-6.39	Gabapentin	171.24	3446	Anticonvulsivants
-6.09	Methamphetamine	149.23	10836	Anorexiants
-6.04	Disulfiram	296.5	3117	Drugs used in alcohol dependence:
-5.94	Levodopa	197.19	6047	Antiparkinson agents
-5.9	Meprobamate	218.25	4064	Miscellaneous anxiolytics, sedatives and hypnotics
-5.63	Phenelzine	136.19	3675	Antidepressants
-5.41	Ethosuximide	141.17	3291	Anticonvulsivants
-5.32	Pregabalin	159.23	5486971	Anticonvulsivants
-5.24	Levetiracetam	170.21	5284583	Anticonvulsivants
-5.18	Caffeine	194.19	2519	CNS stimulants
-4.55	Trimethadione	143.14	5576	Anticonvulsivants
-4.28	Acamprosate	181.21	71158	Drugs used in alcohol dependence:
-4.09	Vigabatrin	129.16	5665	Anticonvulsivants
-3.91	Chloral hydrate	165.4	2707	Miscellaneous anxiolytics, sedatives and hypnotics
-3.57	Divalproex sodium	310.4	23663956	Anticonvulsivants
-3.55	Valproic acid	144.21	3121	Anticonvulsivants
-3.28	Sodium oxybate	126.09	23663870	Miscellaneous anxiolytics, sedatives and hypnotics
